# *MECP2* duplication phenotype in symptomatic females: report of three further cases

**DOI:** 10.1186/1755-8166-7-10

**Published:** 2014-01-28

**Authors:** Francesca Novara, Alessandro Simonati, Federico Sicca, Roberta Battini, Simona Fiori, Annarita Contaldo, Lucia Criscuolo, Orsetta Zuffardi, Roberto Ciccone

**Affiliations:** 1 Department of Molecular Medicine, University of Pavia, Pavia, Italy; 2 Medical School Department of Neurological and Movement Sciences-Neurology (Child Neurology and Psychiatry), University of Verona, Verona, Italy; 3 Department of Developmental Neuroscience, IRCCS Stella Maris, Calambrone, Pisa, Italy; 4 National Neurological Institute C. Mondino, Pavia, Italy

**Keywords:** *MECP2*, Xq28 duplication, X chromosome inactivation

## Abstract

**Background:**

Xq28 duplications, including *MECP2* (methyl CpG-binding protein 2; OMIM 300005), have been identified in approximately 140 male patients presenting with hypotonia, severe developmental delay/intellectual disability, limited or absent speech and ambulation, and recurrent respiratory infections. Female patients with Xq28 duplication have been rarely reported and are usually asymptomatic. Altogether, only fifteen symptomatic females with Xq28 duplications including *MECP2* have been reported so far: six of them had interstitial duplications while the remaining had a duplication due to an unbalanced X;autosome translocation. Some of these females present with unspecific mild to moderate intellectual disability whereas a more complex phenotype is reported for females with unbalanced X;autosome translocations.

**Findings:**

Here we report on the clinical features of three other adolescent to adult female patients with Xq28 interstitial duplications of variable size, all including *MECP2* gene.

**Conclusions:**

Mild to moderate cognitive impairment together with learning difficulties and speech delay were evident in each of our patients. Moreover, early inadequate behavioral patterns followed by persistent difficulties in the social and communication domains, as well as the occurrence of mild psychiatric disturbances, are common features of these three patients.

## Background

Over the last decade, Xq28 duplications including *MECP2* (methyl CpG-binding protein 2; OMIM 300005), have been identified in approximately 140 male patients presenting with hypotonia, severe developmental delay/intellectual disability (DD/ID), limited or absent speech and ambulation, and recurrent respiratory infections [1-25]. Female patients with Xq28 duplication have been rarely reported and are usually asymptomatic. Highly skewed X-chromosome inactivation (XCI) with preferential inactivation of the duplication-bearing X chromosome has been usually demonstrated in their blood samples [26]. Until recently, fifteen symptomatic females with Xq28 duplications including *MECP2* had been reported. Six of them presented with interstitial duplications while in the remaining cases the duplication originated by an X;autosome translocation [3,20,24,25,27-32].

Some of these females present with unspecific mild to moderate intellectual disability whereas a more complex phenotype is reported in subjects with unbalanced X;autosome translocations (Table 1).

**Table 1:** **A summary of genetic and clinical features of symptomatic female patients with *MECP2 *** **duplication reported so far in literature and including the three cases reported herein (Group A: patients with small interstitial Xq28 duplication, Group B: Xq28 duplication due to X;autosome translocations)**

		**Group A**	**Group B**
Age		7-21 years	18 months-19 years
Genetic features			
Duplicate segment length		107.5 Kb-700 Kb	0.29 Kb-16.6 Mb (for two cases the size is unknown but cytogenetic visible)
Inheritance	*de novo*	4	8
	Maternal	5	0
	Unknown	0	1
XCI	Random	6	2
	Skewed	3	1
	Unknown	0	6
Clinical features			
Abnormal general conditions		5/9 (55%)	9/9 (100%)
Dysmorphic patterns		3/9 (33%)	9/9 (100%)
Delayed motor development		4/9 (44%)	9/9 (100%)
Abnormal language development		6/9 (67%)	9/9 (100%)
Intellectual disability		7/9 (78%)	9/9 (100%)
Mood and behaviour	Affected	4/9 (44%)	1/9 (11%)
	Unknown	5/9 (55%)	8/9 (88%)
Social conduct	Affected	8/9 (88%)	0/9 (0%)
	Unknown	1/9(11%)	9/9 (100%)
Autistic features		4/9 (44%)	0/9 (0%)
Seizures		1/8 (13%)	3/9 (33%)
Brain MRI	Abnormal	0/9 (0%)	3/9 (33%)
	Unknown	1/9 (11%)	6/9 (67%)

Abnormal general conditions: growth retardation, constipation, hypotonia and/or joint laxity.

Dysmorphic pattern: microcephaly, trigonocephaly, facial dysmorphisms, multiple skeletal and/or organs dysmorphisms.

Language development: either delayed or impaired during school-age and adolescence.

Intellectual disability: Group A: either mild or moderate; Group B: severe only.

Brain-MRI: cortical atrophy along with white matter involvement.

Here we report on the clinical features of three other adolescent to adult females patients with Xq28 interstitial duplications of variable size, all including *MECP2* gene, in order to improve the knowledge about phenotype associated with this clinical condition.

## Clinical report

**Figure 1:**
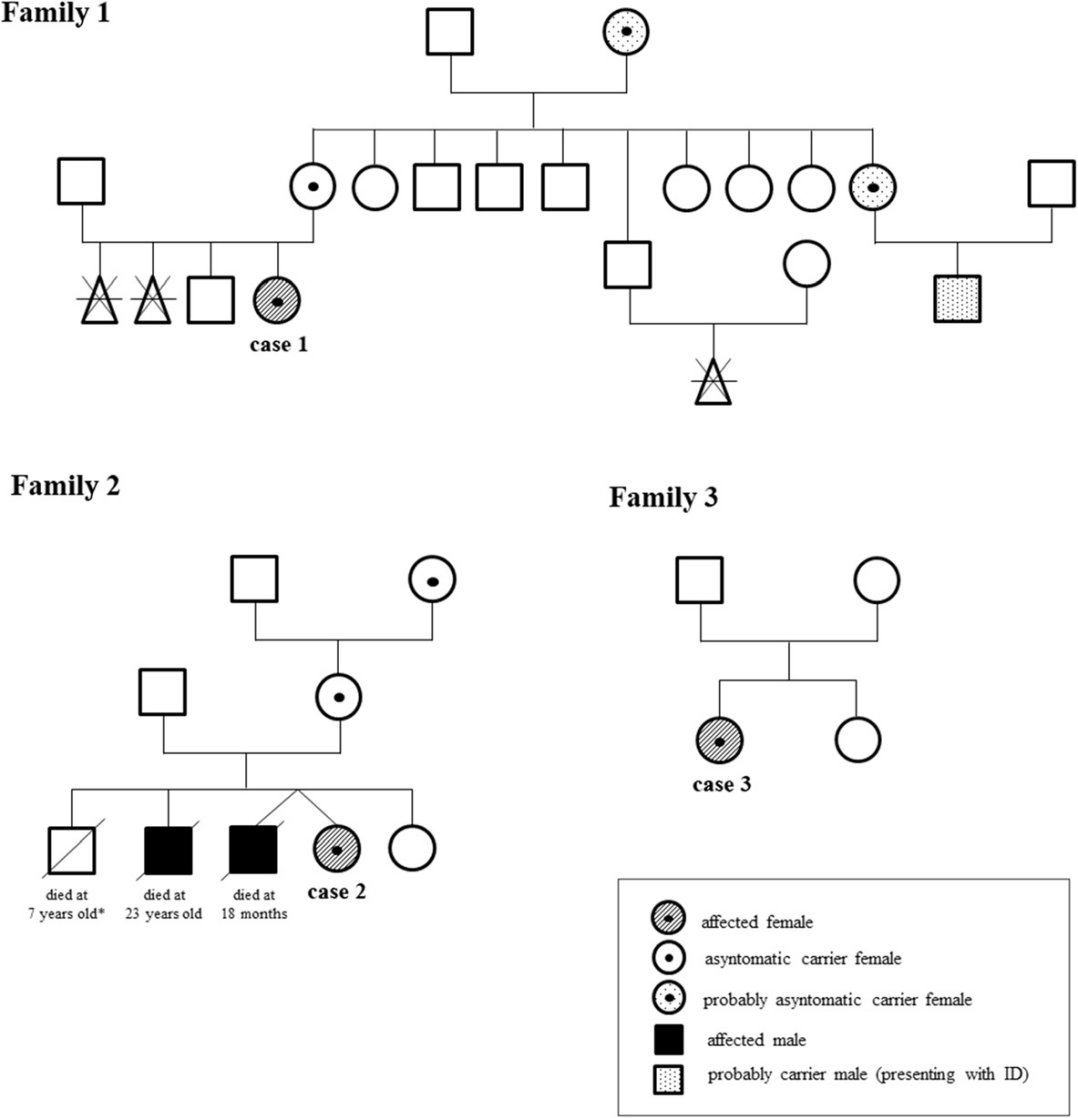
**Pedigree of the three families with clinical details for carriers and patients.** The male who died at 23 years of age in family 2 has been previous published (family 10) in Clayton-Smith et al., 2009 [9]. The healthy male in family 2 died at the age of 7 years old because of an accident.

Two out of three patients reported in this paper had a positive family history: intellectual disability (ID) was present in different male relatives of cases 1 and 2. The male twin and the elder brother of case 2 were affected by severe psychomotor delay/intellectual disability and died at 18 months and 23 years of age respectively because of respiratory infections; facial dysmorphisms were present in both. The three pedigrees together with the clinical details for the carriers and patients are reported in Figure 1.

Gestation and delivery were unremarkable in all 3 cases; both pre- and post-natal growth were normal.

Mild signs of early psychomotor retardation and speech delay were recorded in all children by their infancy while feeding and sleep disturbances have been reported since 18 months of age in case 3.

By puberty, mild motor clumsiness was evident in all of them with scarce motor fluidity and mild disturbances of coordination along with hypotonia and joint laxity.

Presently, cognitive functions were affected in all patients. Speech development was markedly delayed in case 1; mild delay was observed in the remaining two. Learning difficulties were present in all. Mild to moderate ID was evident in all patients after intelligence testing administration at different ages (ID was ascertained according to WISC-R scale). In particular case 3 revealed a decline of IQ from borderline at 6 years of age to mild intellectual disability when she was 17 years old. Deficit in adaptive behaviour with early behavioural disturbances were evident in all cases (Table 2). Interestingly, behavioural patterns differed among the patients, both qualitatively and temporally. In case 1 tendency to social withdraw was present in pre-school age lasting until adolescence. Conversely, hyperactivity with attention difficulties were characteristic of cases 2 and 3; some anxiety features get started during adolescence in case 2, whereas depressive mood along with anxious traits came out at the same age in case 3. Social conduct was normal in case 1, it was getting worse over time in case 2 whereas it was always impaired in case 3. None of them showed stereotyped or repetitive behaviours.

**Table 2:** Major clinical findings for all three reported patients

	**Case 1 (14 years)**	**Case 2 (21 years)**	**Case 3 (19 years)**
	- two spontaneous abortions	- male twin: facial dysmorphism severe early delay; death at 18 months of age	
	- one maternal uncle with ID	- one brother: facial dysmorphism severe developmental delay, seizures, autistic features, death at 23 years of age	
**Physical data**			
(at birth)			
GW	40	39	40
L (cm)	49 (50 pc)	Not known	51 (50 pc)
BW (g)	2400 (10 pc)	3000 (50 pc)	3780 (75 pc)
HC (cm)	36 (50 pc)	Not known35	(50 pc)
**Early development**			
Walking (months)	18	13	15
Speech onset (years)	5 (few words) → 12 (fair)	4	3
Behaviour	Difficulty of separation and isolation	Hyperactivity	Tantrum
**School age**			
Neurological condition	Normal	Normal	Normal
Learning	Difficulties	Difficulties	Difficulties
Intelligence	Moderate (IQ 41)	Borderline (IQ NA)	Borderline (IQ 80 when 6 years)
Communication/social conduct	Difficulties	Good	Difficulties (since 3 years)
Behaviour	Quiet and withdrawn	Hyperactivity, attention deficit, impulsiveness	Hyperactivity
**Adolescence**			
Neurological condition			
Motor coordination	Fair	Poor	Fair
Hypotonia/joint laxity	Yes	Yes	Yes
Intelligence	Scarce (IQ NA)	Borderline (IQ 84 when15 years)	Mild (IQ 57 when17 years)
Communication/social conduct	Difficulties	Difficulties	Difficulties
Behaviour	Quiet and meek	Hyperactive, anxious, mood disorder	Anxious, depressive mood
**Dysmorphic features**	Minor facial dymorphisms (broad nasal bridge, prognatism, mild ocular hypertelorism)	Minor facial dysmorphisms (broad nasal bridge, prognatism)	None
**Diagnostics**			
EEG	Normal	NA	Posterior slow waves
MRI	Normal	NA	Normal

GW: gestational age; L: length; BW: body weight; HC: head circumference; IQ: intelligence quotient (ascertained according to WISC-R scale); ID: intellectual disability; NA: not ascertained.

**Figure 2:**
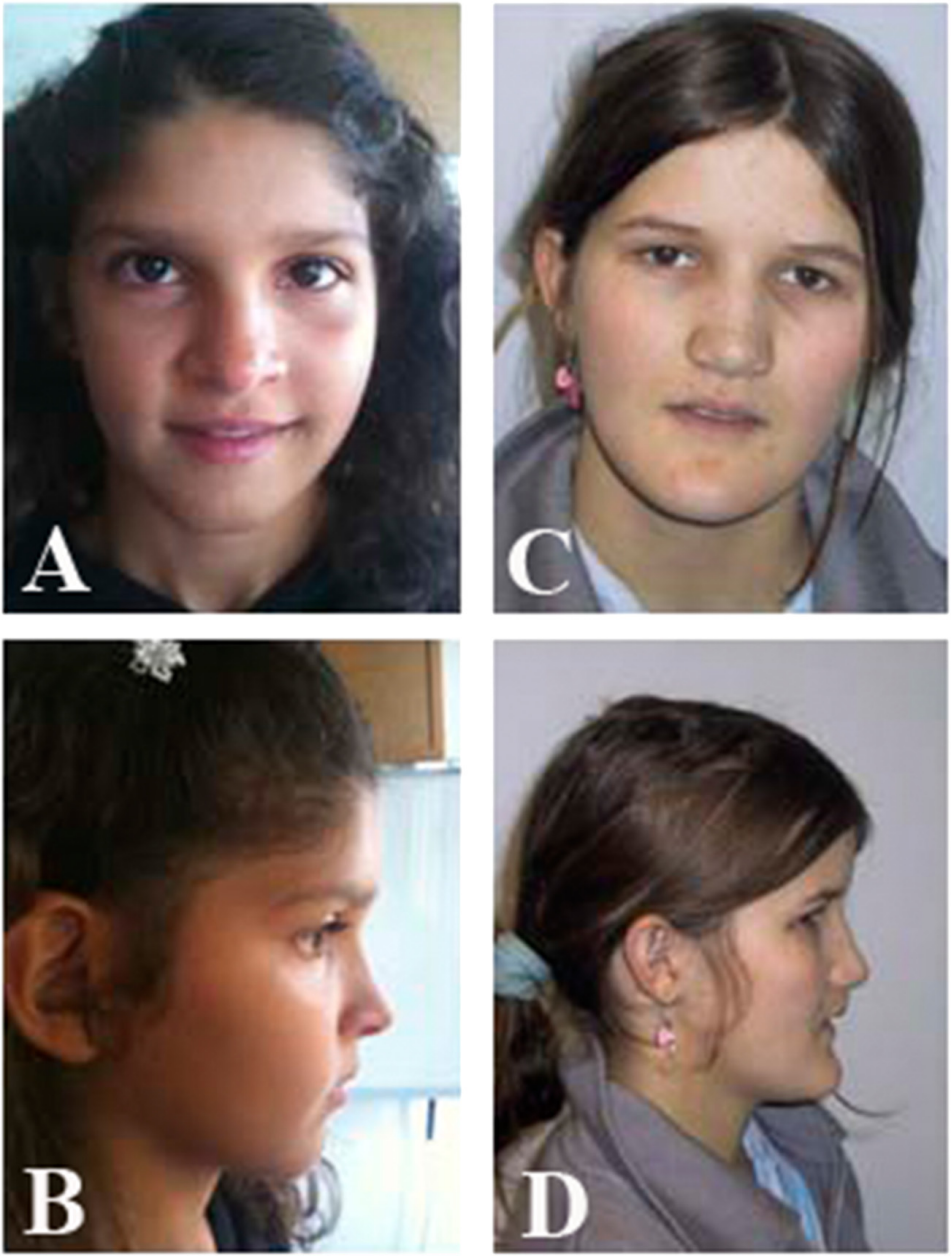
Pictures of case 1 when she was 14-years-old (A and B) and case 2 at 21 years of age (C and D).

Minor facial dysmorphic features were observed only in cases 1 and 2: broad nasal bridge, prognatism, mild ocular hypertelorism (Figure 2).

EEG was normal in case 1, whereas posterior slow waves were recorded in case 3. Head-brain MRI imaging (cases 1 and 3) was normal. EEG and brain MRI were not available in case 2.

Major clinical findings for all three patients are summarized in Table 2.

Informed consent was obtained for all patients. Parents of cases 1 and 2 gave permission for their photo publication.

## Results

**Figure 3:**
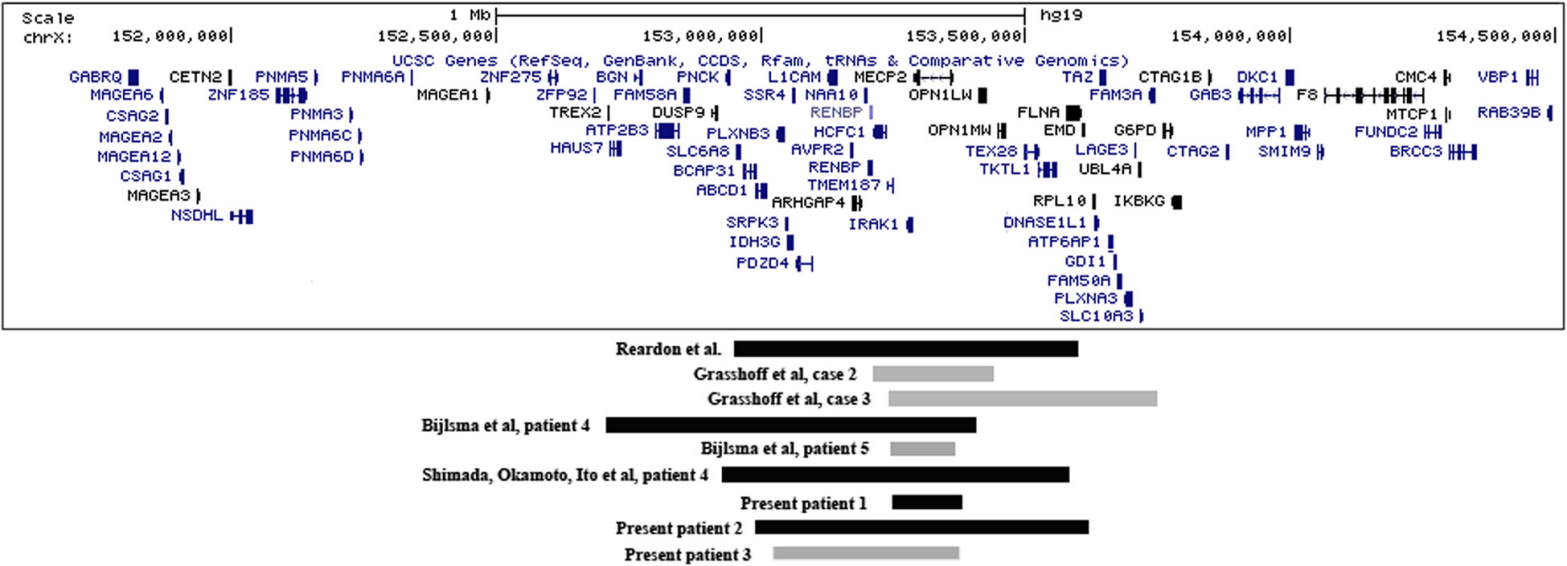
**Schematic representation of Xq28 region.** All nine female patients with intrachromosomal Xq28 duplication are represented. Familiar cases are in black, while *de novo* duplications in grey. Gene content of the region is shown from the UCSC Genome Browser version Human February 2009 (hg19).

The array-CGH showed a Xq28 duplication in all the three female patients. Case 1: arr[hg19] Xq28(153,238,518-153,406,174)×3 mat, case 2: arr[hg19] Xq28(152,987,955-153,609,113)×3 mat and case 3: arr[hg19] Xq28(chrX:153,015,806-153,405,806)×3 dn (probe positions are referred to hg19) (Figure 3).

The duplications presented different sizes: 167 Kb, 621 Kb and 390 Kb respectively. In all the cases *MECP2* and *IRAK1* were included in the duplication.

X chromosome inactivation test performed on DNA extracted from blood and saliva showed a random X inactivation in case 1 and in her mother, who carried the same duplication (case 1: R2/R1 = 3.1 and 2.7 in blood and saliva respectively; mother: R2/R1 = 2 and 1.8 in blood and saliva respectively). Case 2 was also tested together with her mother and her maternal grandmother who were both healthy carriers of the Xq28 duplication. The test showed a 100% skewed inactivation in blood but a random inactivation in DNA extracted from buccal swabs in all of them (R2/R1 = 0.39, R2/R1 = 0.45, R2/R1 = 0.6 for case 2, her mother and her grandmother respectively). In case 3 the test showed a skewed X chromosome inactivation both in blood and saliva (R2/R1 = 14,7 and 11,7 respectively). In this last case microsatellite analysis demonstrated that the anomaly originated *de novo* on the paternal X chromosome (data not shown).

## Discussion

*MECP2* gene, located on chromosome Xq28, encodes for an essential epigenetic regulator of postnatal brain development [33]. Loss-of-function *MECP2* mutations had been identified as the cause of Rett syndrome, mainly affecting females [34] and initially thought to be lethal in males [22]. Duplications involving *MECP2* have been frequently identified in male patients presenting with a different phenotype from that of Rett syndrome and mainly characterized by severe mental retardation, recurrent respiratory infections, epilepsy, cerebellar degenerative change and progressive encephalopathy [4-6,8,12,14,24]. Whereas the phenotypic effect of *MECP2* duplications has already been well documented in males, the clinical implications determined by these rearrangements is still poorly known in females. So far, females with both small intrachromosomal duplications including *MECP2*[20,24,29,32] (Figure 3) and large Xq28 duplications resulting from an unbalanced X;autosome translocation have been documented [3,25,27,28,30-32]. In the latter cases (Table 1, group B cases) a more severe phenotype is present: intellectual disability, abnormal language development and delayed motor development is present in all of them. Moreover epilepsy and abnormal brain MRI are reported in 33% of X;autosome translocation cases, while autistic features are absent in all of them. This more severe phenotype is likely to be due to the partial monosomy for the portion of the autosome where the Xq was transposed.

Thus, *MECP2* is the primary dose-sensitive and critical gene responsible for neurological phenotypes both in males and females. In male mice, the overexpression of Mecp2 causes a progressive neurological disorder, including motor dysfunction, hypoactivity, tremor, ataxia, and premature death; ubiquitous elevated expression also led to lethal heart and skeletal malformations [35,36].

Here we described three further female patients carrying a submicroscopic Xq28 duplication involving *MECP2* (Figure 3)*.* Two of them carried a familial Xq28 duplication, but in both cases their mothers did not present any pathological phenotype. Differences in XCI patterns have been hypothesize as responsible for the different phenotypes and outcome in this chromosome imbalance. It is reasonable to assume that the random XCI is the crucial point leading to the intellectual disability phenotype in symptomatic patients, whereas a highly skewed XCI with a preferential inactivation of the duplicated X chromosome is a protective factor.

Our experiments to test the X chromosome inactivation did not reveal any difference between mother and daughter in cases 1 and 2. Those data did not explain the phenotypic differences among female carriers of Xq28 duplication both in cases 1 and 2. However, it is likely that in symptomatic patients the duplicated chromosome X was preferentially active during the critical stages of embryonic development thus impairing the normal brain development. Moreover, we tested only blood and saliva and we could not determine the X inactivation status of other tissues.

Despite the different sizes of the identified duplicated regions, *IRAK1* was included within each of them. Duplication of this gene has been hypothesized to be responsible for the severe and recurrent respiratory infections in duplicated males [13]. This feature, that is the main cause of death in males, has been rarely reported in female patients [24 patient 1,28,29 patient 1,32 patients 1, 2, 3 and 5] and was absent in all the three cases reported herein.

Mild to moderate cognitive impairment together with learning difficulties and speech delay were evident in each of our patients. It is noteworthy that the patient 3, with *de novo* microduplication, presented with an apparent disease progression, as shown by the mildly declined score of her cognitive abilities (see Table 2). Similarly *MECP2* mutations in Rett syndrome females lead to a gradual loss of acquired skills around the first year of age [37], associated with postnatal microcephaly, a feature not present in *MECP2* duplicated females.

In the only patient (case 3) with *de novo* Xq28 microduplication, the anomaly originated on the paternal X chromosome. Similarly, a paternal origin was demonstrated in other two females with *de novo* Xq28 interstitial duplications [32] in agreement with the X chromosome vulnerability at male meiosis [38].

Early inadequate behavioral patterns followed by persistent difficulties in the social and communication domains, as well as the occurrence of mild psychiatric disturbances are common features of these three patients. Hyperactivity with attention deficit was reported in 2 out of three patients during school age, whereas symptoms such as anxiety, or a depressive mood developed during the adolescence. The occurrence of behavioural changes, as well as the onset of psychiatric disturbances during the adolescence, is a common event in children affected by mild difficulties (such as our three cases) and it can be related to their social difficulties. None of them developed autistic features as described in other female patients affected by a similar condition [29, 32 case 4].

## Conclusion

In conclusion we could not highlight the reason for differences in the phenotypes of *MECP2* duplicated females: neither the size of the duplication nor the X-inactivation pattern as assessed in blood or other easy available tissues, may predict the outcome of these subjects. This highly variable clinical presentation makes genetic counseling difficult in terms of prognosis, especially in prenatal cases.

## Methods

Written informed consent was obtained from the parents of the patients for publication of this manuscript and any accompanying images.

### Molecular karyotyping

Molecular karyotyping was performed by using the Agilent array 180 K (Human Genome CGH Microarray, Agilent Technologies, Santa Clara, CA, USA) for all patients according to the manufacturer’s protocol. Data analysis was performed using Agilent Genomic Workbench Standard Edition 6.5.0.58.

Probe positions are referred to hg19.

### X-inactivation test

The X-chromosome inactivation (XCI) patterns were analyzed for all patients by genotyping the CAG repeats in the androgen receptor gene. 1 μg of DNA (extracted from blood or saliva) was digested at 37°C overnight with HpaII restriction enzyme (New England Biolabs, Beverly, MA, USA) in a total volume of 50 μl containing 5 μl buffer 10× and 2 μl of enzyme (5 U/μl). After digestion the enzyme was inactivated at 65°C, and PCR was performed on 200 ng digested/undigested DNAs with specific primers for HUMARA locus. One of the primer set was labeled with the fluorescent dye (FAM). The PCR mixture contained 10× Taq reaction buffer (200 mM Tris pH 8.4, 500 mM KCl), 50 mM MgCl2, dNTPs (2 mM), primers (20 μM), 2.5 units of Taq polymerase (Invitrogen, Carlsbad, CA, USA) for a final volume of 50μl. Amplification was carried out in a temperature cycler with the following PCR cycling profile: preheating at 95°C for 5 min, followed by 95°C for 45 sec, 60°C for 30 sec, 72°C for 30 sec for 28 cycles, and a final extension at 72°C for 7 min. The PCR samples were loaded onto a 2% agarose gel, stained with ethidium bromide, and directly visualized under ultraviolet (UV) illumination. Analysis of fluorescent samples was performed using ABI 3100 Genetic Analyzer (Applied Biosystems).

Because CAG repeats site and the methylation sites of the androgen receptor gene were included in the PCR fragments, PCR products could only be obtained from the undigested DNA derived from the inactive X-chromosome. Analysis of PCR amplicons was performed using ABI 3100 Genetic Analyzer (Applied Biosystems) to determine the PCR product size and obtain peak areas. Finally, the XCI patterns were classified as random (a ratio higher than 20:80 and lower than 80:20) or skewed (higher than 80:20 or lower than 20:80) according to Allen et al., 1992 [39].

### Microsatellite analysis

Genotyping of polymorphic loci in the patients with *de novo* Xq28 duplications and in her parents was performed by amplification with primers labeled with fluorescent probes (ABI 6-Fam and 8-Hex), followed by analysis on ABI 3100 Genetic Analyzer (Applied Biosystems, Foster City, CA). Primers were designed using the database tool Tandem Repeats Finder (http://tandem.bu.edu/trf/trf.intermediate.submit.html).

## Abbreviations

MECP2: Methyl CpG-binding protein 2; ID: Intellectual disability; EEG: Electroencephalogram; MRI: Magnetic resonance imaging; PCR: Polymerase chain reaction; array-CGH: Array comparative genomic hybridization; XCI: X-Chromosome inactivation.

## Competing interests

The authors declare that they have no competing interests.

## Authors’ contributions

FN carried out all the molecular cytogenetic analysis and drafted the manuscript. AS, FS, RB, SF, AC and LS collected all clinical data, conceived the work, and participated in its design, drafted and revised the manuscript. OZ and RC conceived the work, and participated in its design, drafted and revised the manuscript. All authors analyzed all results and read and approved the final manuscript.
